# Factors Associated with Initiation and Sustenance of Stress Management Behaviors in Veterinary Students: Testing of Multi-Theory Model (MTM)

**DOI:** 10.3390/ijerph17020631

**Published:** 2020-01-18

**Authors:** Vinayak K. Nahar, Julia K. Wells, Robert E. Davis, Elizabeth C. Johnson, Jason W. Johnson, Manoj Sharma

**Affiliations:** 1 Department of Dermatology, University of Mississippi Medical Center, Jackson, MS 39216, USA; 2 Department of Preventive Medicine, School of Medicine/John D. Bower School of Population Health, University of Mississippi Medical Center, Jackson, MS 39216, USA; 3 Center for Animal and Human Health in Appalachia, College of Veterinary Medicine, Lincoln Memorial University, Harrogate, TN 37752, USA; julia.wells@lmunet.edu (J.K.W.); elizjohnson423@gmail.com (E.C.J.); jason.johnson02@lmunet.edu (J.W.J.); 4 Substance Use and Mental Health Laboratory, Department of Health, Human Performance and Recreation, University of Arkansas, Fayetteville, AR 72701, USA; red007@uark.edu; 5 Department of Behavioral & Environmental Health, School of Public Health, Jackson State University, Jackson, MS 39217, USA; manoj.sharma@jsums.edu

**Keywords:** stress, relaxation behavior, multi-theory model, health behavior change

## Abstract

Veterinary students across the United States face the challenge of stress during school every day. When managed improperly, stress can become chronic and manifest in physical and emotional consequences. The purpose of this study was to examine the utility of the multi-theory model (MTM) of health behavior change in predicting the initiation and sustenance of stress management behaviors among veterinary students. A cross-sectional design was used to study the efficacy of the MTM in predicting initiation and sustenance of stress management behaviors among veterinary students at a private College of Veterinary Medicine in the Southeast United States. Researchers collected data using a 54-item valid and reliable survey. Only students who did not already engage in daily stress management behaviors were included in the study. After recruitment and exclusion, a total of 140 students remained and participated in the study. Hierarchical multiple regression revealed that, for initiation of stress management behaviors, 49.5% of the variance was explained by depression, academic classification, and behavioral confidence. Regarding sustenance of stress management behaviors, 50.4% of the variance was explained by perceived stress, depression, academic classification, and emotional transformation. MTM serves as a promising framework for predicting initiation and sustenance of health behavior change. Based on the results of this study, interventions aimed to promote stress management behaviors in veterinary students should focus on the MTM constructs of behavioral confidence and emotional transformation.

## 1. Introduction

Stress is defined as a psychosocial and biochemical human reaction to situations that may elicit feelings of anxiety or fear [1]. In moderate amounts, sometimes stress can be beneficial and even promote learning opportunities among students [2]. For example, a moderate amount of stress to perform well on an exam may encourage students to spend more time studying. However, extreme amounts of exposure to stressors or misjudged perception of stressors may become debilitating, causing a wide range of negative consequences on the affected individual [2]. Negative consequences of stress manifest in both emotional and physical changes. Emotional effects include anxiety, anger, irritability, and depression [1,2,3]. Physical consequences of stress include weight loss, changes in sleeping habits, increased heart rate, difficulty breathing, and digestive problems [1,2,3]. Furthermore, stress has been identified as a risk factor for numerous chronic conditions, including heart disease, cancer, respiratory illness, and cirrhosis of the liver [4].

The concept of stress is widely prevalent in veterinary education and veterinary medical clinics worldwide [5,6]. Program rigor, competition among peers, emotional fatigue, and high debt load faced by graduates all contribute to a stressful environment throughout veterinary schools [7]. Studies have shown that nearly 38% of veterinary students each year report feeling depressed [5]. This statistic is high when compared to non-veterinary student populations [8]. After veterinary school, in the veterinary medical field, stress levels remain high. Since leaving their educational programs, studies show that 14.4% of male and 19.1% of female veterinarians report having considered suicide due to emotional fatigue and depression caused by workplace stress [9]. Chronic or persistent stress in veterinary school can have severe impacts on student learning by interfering with concentration, decision-making ability, and emotional wellbeing [5]. Veterinary academic institutions must address the problem of stress among students to effectively promote quality learning and prevent negative consequences of stress, such as elevated suicide rates, post-graduation.

Perhaps the simplest solution to the problem of stress among veterinary students is stress-management training or teaching anxiety-reducing behaviors to students during school. This could be performed via numerous mechanisms—utilizing school counselors, external specialists, or even outreach programs orchestrated within the student body [10,11,12]. One study found evidence that practicing a mindfulness intervention as an anxiety-reducing behavior may temporarily lower stress levels in veterinary students [11], and another found that encouraging a growth mindset, instead of a fixed mindset, helps students to appropriately handle stressful situations [12]. While these strategies appear promising, studies have also found that veterinary students are often reluctant to seek counseling and medical attention for mental health problems, possibly due to a negative stigma associated with mental health issues [13]. This reluctance to seek help makes anxiety-reducing interventions difficult to implement at veterinary schools. Additionally, researchers have recommended various socio-behavioral models for promoting behavior changes among students. The health belief model, transtheoretical model, and PRECEDE–PROCEED model are three of the most commonly recommended models [14]. However, these models are not adequate for promoting health behavior change among veterinary students specifically for multiple reasons. The health belief model focuses more on short-term behavior change than long-term change. Given the problems with continued stress and elevated suicide rates after school, a long-term focus is essential for interventions with veterinary students. The transtheoretical model is not specific toward health education, making it less suitable for use in anxiety-reducing behaviors. Finally, the PRECEDE–PROCEED model is too broad to be pragmatic for application and testing in veterinary practice [14,15].

Recently, Sharma has taken note of the limitations of previous health behavior theoretical models and proposed a fourth-generation theoretical model called the Multi-Theory Model of Health Behavior Change (MTM) [14,16]. The MTM model incorporates multiple different socio-behavioral theories, highlighting the strengths of each and bringing them together into a model of two components—initiation and sustenance of health behavior change. This model has already been tested on college student populations regarding the prediction of initiation and sustenance of health behavior changes such as a reduction in binge-drinking, increase in eating fruits and vegetables, improvement in sleep behaviors, increased physical activity, and portion size control [17,18,19,20,21]. Ideally, the MTM should work well among veterinary students for predicting initiation and sustenance of health behavior change because it is both specific for health education and sustainable for long-term change. Within this backdrop, the objective of this study was to examine the utility of the MTM in predicting the initiation and sustenance of stress management behaviors among veterinary students.

As mentioned previously, the MTM model of health behavior change is based on two interlinking components: initiation (i.e., one time) and sustenance (i.e., continuation) of health behavior change. Each of the two components, initiation and sustenance, are composed of three unique constructs. The constructs for initiation of health behavior change are as follows: participatory dialogue, behavioral confidence, and changes in the physical environment. Participatory dialogue involves two-way communication between a health educator and a subject that focuses on the advantages and disadvantages of initiating a health behavior change. Behavioral confidence, named on the basis of its simplicity and cultural specificity, focuses on a subject’s confidence to change health behavior in the future specifically, not in present day. Finally, “changes in the physical environment” construct emphasizes that the subject must modify his/her physical environment to make resources that support health behavior change more readily available. The constructs for sustenance of health behavior change are the following: emotional transformation, practice for change, and changes in the social environment. Emotional transformation involves focusing one’s feelings and emotions on health behavior change and channeling thoughts toward sustaining the change. Practice for change focuses on reflective actions, during which the subject thinks about his or her health behavior change. One mechanism in which subjects may embody practice for change is by using a journal or diary daily to track progress. Lastly, the construct “changes in the social environment” involves surrounding oneself with a firm support system that encourages health behavior change. Using these six constructs, empirical support thus far in support of the MTM leads us to believe that one can successfully initiate and sustain a health behavior change regarding stress-management behaviors [14].

## 2. Materials and Methods

### 2.1. Participants and Procedure

Researchers utilized a cross-sectional study design to investigate the efficacy of the MTM model in predicting initiation and sustenance of anxiety-reducing behaviors among veterinary students at a private University in the Southeast United States. Independent study variables were the previously described MTM constructs, while dependent variables were the intention to initiate and the intention to sustain relaxation behaviors. Students were eligible to participate in the study if they were currently enrolled veterinary students at the University and were not already engaging in twenty minutes or more of relaxation behavior per day. Due to the nature of this study focusing on health behavior change, students already participating in relaxation behaviors were excluded from the study. Following initial recruitment, three hundred and forty-two students of veterinary medicine consented to participate in the current study. After exclusion of those currently engaging in intentional relaxation behaviors, 140 students remained and were included as participants of this study.

Data collection occurred via a 54-question online survey administered to participants through email, and collection took place over a three-week period with two reminder emails. All participants were over the age of 18 and gave informed consent before completing the survey. This study was granted ethics approval by institutional review board (IRB) at the Lincoln Memorial University (protocol number: 661 V.0).

### 2.2. Instrumentation

The survey instrument consisted of 54 items. The first 10 items assessed students’ current perceived stress levels using the perceived stress scale [22], with questions such as “In the last month, how often have you felt nervous and ‘stressed’?” The next 4 items assessed Anxiety and depression using the four-item patient health questionnaire (PHQ-4) [23]. Items 15–23 identified demographic characteristics such as age and marital status, and item 24 determined eligibility by asking about current relaxation behavior practice. The remaining 30 items focused on the MTM constructs for both initiation and sustenance of relaxation behavior. Advantages and disadvantages of participatory dialogue were gauged with 5 items each, all of which were scored on a 5-point scale. The disadvantage score was subtracted from the advantage score to give a final score for the construct. Behavioral confidence was gauged with 5 items focused on confidence in practicing relaxation behavior despite various challenges (“being busy”, “not enjoying it”, etc.), and 3 items gauged changes in the physical environment in a similar manner. The emotional transformation was gauged using 3 items, such as “How sure are you that you can motivate yourself to practice relaxation for 20 min daily?” Items 47–49 assessed practice for change by asking about keeping a journal and adjusting plans to make time for relaxation behavior. “Changes in the social environment” construct was gauged using 3 items as well with questions such as “How sure are you that you can get the help of a friend to support you with practicing relaxation for 20 min daily?” Items 53 and 54 assessed the initiation and sustenance, respectfully, by asking “How likely is it that you will practice relaxation for 20 min daily in the upcoming week?” (53) and “How likely is it that you will practice relaxation for 20 min daily from now on?” (54). For all construct questions, answered were scored on a scale of 0–5, with higher scores associated with higher likelihood of initiation/sustenance of behavior change.

Face, content, and construct validity were all established for the survey instrument used in the study. Face and content validity were determined by utilizing six experts in the field, while construct validity was determined using confirmatory analysis with the maximum likelihood method. The maximum likelihood method gave 1-factor solutions for each subscale, all of which matched criteria of factor loadings over 0.32 and Eigen values over 1.0. Cronbach’s α was used to establish internal consistency of the survey instrument, with acceptable reliability denoted as a Cronbach’s α value of ≥0.70 [24].

### 2.3. Data Analyses

Descriptive statistics were performed for all variables. The dependent variables, intention to initiate and to sustain relaxation behaviors, were calculated on a continuous scale. To assess statistically significant relationships between demographic covariates and MTM variables of interest, Pearson Product-Moment correlations were performed for continuous variables and independent samples t-tests were performed for categorical variables. Analyses were performed to determine the utility of MTM in predicting intention to both initiate and to sustain relaxation behaviors in two separate models, model 1 and model 2. In model 1, initiation, independent variables were participatory dialogue, behavioral confidence, and changes in the physical environment. In model 2, sustenance, independent variables were emotional transformation, practice for change, and changes in the social environment. For both models, researchers first determined statistically significant demographic covariates and entered them into block 1. Hierarchical multiple regression was then performed among the significant covariates and the independent variables (MTM constructs, entered into block 2) for each model. All statistical analyses of data were completed using IBM SPSS statistical software version 25.0 with a significance level of 0.05.

## 3. Results

### 3.1. Participants

The vast majority of participants were females (92.9%), with approximately 88% identifying as White/Caucasian, Table 1. Little representation by racial minorities was observed in this study. As this study was conducted in rural Appalachia, this low representation of non-White ethnic groups may not be surprising. The majority of participants were second-year students (30.7%), followed by third-year (26.4%), first-year (25.0%), and fourth-year (17.9%). Participants generally held high grade point averages with more than 70% having a GPA (grade point average) above 3.0, which is not uncommon among medical programs. Furthermore, most participants were not married (80.0%), reported no children (95.7%), were not employed (88.6%), and resided off campus (95.0%). The average stress level of the participants was 21.64 (SD: 6.49, possible range 0–40). Sixty percent of the participants screened positive for anxiety, and 32.9% screened positive for depression.

### 3.2. Multi-Theory Model and Intentional Relaxation Behavior

To examine the relationship between MTM constructs and the initiation and sustenance of relaxation behaviors, hierarchical multiple regression models were constructed. Step one of these regressions controlled for the variables perceived stress, depression, and academic classification, as each displayed significant bivariate relationships with both initiation and sustenance of relaxation behavior. Descriptive statistics and reliabilities for specific MTM variables can be seen in Table 2.

### 3.3. Initiation of Intentional Relaxation Behavior

For initiation of intentional relaxation behavior, 15.9% of the variance in initiation was explained by the lower-order terms forming the base model (*R*^2^ = 0.159, *p* < 0.001), Table 3. Herein, significance was observed for depression (*b* = −0.413, *p* = 0.040) and academic classification (*b* = −0.597, *p* < 0.001). Variance accounted for increased to 49.5% with the inclusion of MTM constructs in model 2 (*R*^2^ = 0.495, *p* < 0.001). In the final model, only behavioral confidence exhibited a significant association with initiation of relaxation behavior (*b* = 0.138, *p* < 0.001).

### 3.4. Sustenance for Intentional Relaxation Behavior

For sustenance, lower- order terms perceived stress, depression, and academic classification explained 16.6% of the variance in sustenance for intentional relaxation behavior (*R*^2^ = 0.166, *p* < 0.001), with perceived stress (*b* = −0.035, *p* = 0.009) and academic classification (*b* = −0.454, *p* = 0.001) exhibiting significance, Table 4. MTM constructs were entered in model 2 and provided a substantial increase over the base model in variance accounted for (Δ*R*^2^ = 0.337). The final model explained 50.4% of the variance in sustenance of intentional relaxation behavior (*R*^2^ = 0.504, *p* < 0.001). Once all variables were accounted for in the model, emotional transformation held the only significant relationship to sustenance (*b* = 0.178, *p* < 0.001).

## 4. Discussions

The purpose of this study was to explore the use of the multi-theory model (MTM) of health behavior change in predicting intention for veterinary students to begin consciously performing relaxation behaviors for twenty minutes per day. The MTM is a relatively new, robust model based on two interlinking components: initiation and sustenance of health behavior change. Both initiation and sustenance are composed of three constructs that predict the success of the component. By conducting this study, researchers hoped to identify significant constructs of the MTM that can be used as targets for interventions in veterinary schools to improve the likelihood of student participation in conscious relaxation behaviors.

From the initiation model, significant lower-order predictors included depression (*p* = 0.040) and academic classification (*p* < 0.001), while behavioral confidence was the only significant higher-order construct (*p* < 0.001). The full model predicted 49.5% of the variance in the initiation of conscious relaxation behavior. The construct of behavioral confidence has been derived from Bandura’s self-efficacy theory [25] and Ajzen’s perceived behavioral control theory [26]. It focuses on one’s confidence to initiate a health behavior change despite opposition, a busy schedule, or not enjoying the behavior. Behavioral confidence has been identified as a significant construct in prior MTM studies on different health behavior changes [17,18,19]. One important concept regarding behavioral confidence is that it includes confidence gathered from both internal and external sources, meaning sources such as counselors and mentors are equally as important as inner dialogue and self-regulation. A 2019 study found that the use of a life coach for first-year medical students showed promise for mental health improvements [27]. Veterinary schools could use this idea to promote an intervention targeting behavioral confidence. Weekly meetings with a counselor, mentor, or even small group sessions of classmates could help students to identify barriers to completing relaxation behaviors and work to formulate a plan to prioritize wellness. Potential barriers to student participation in relaxation behaviors include busy class schedules and distractions such as television and social media. Once students identify these barriers and ways to combat them, their behavioral confidence in initiating relaxation behavior should increase. Studies show that veterinary students are often hesitant to seek counseling and help for mental health issues [28,29]; therefore, any proposed interventions should be presented to all students, even those who do not actively seek help. Furthermore, counselors and external sources should encourage students’ own inner-dialogue, as behavioral confidence must come from within the students themselves in addition to external sources.

Regarding the sustenance model, significant lower-order predictors included perceived stress (*p* = 0.009) and academic classification (*p* = 0.001), while emotional transformation was the only significant higher-order construct (*p* < 0.001). The full model predicted 50.4% of the variance in the sustenance of conscious relaxation behavior. The emotional transformation has been identified as a significant construct in past MTM studies on health behaviors such as fruit and vegetable consumption and sleep activity [18,19]. Derived from the self-motivation construct of emotional intelligence theory [30], emotional transformation focuses on overcoming self-doubt and directing one’s emotions and motivation toward the goal of health behavior change, in this case, participating in twenty minutes of conscious relaxation behavior per day. Studies show that veterinary students often experience low self-esteem, especially during the first year [31]. This may explain the significance of the emotional transformation construct, as low self-esteem suggests an inability to overcome self-doubt. As with behavioral confidence, interventions in the form of weekly meetings with counselors, mental health educators, and/or small group sessions could be the best way to target the construct of emotional transformation for the sustenance of conscious relaxation behaviors. During these sessions, educators should discuss self-motivation with students as well as the concept of a growth mindset. While the construct of emotional transformation has not specifically been studied in regards to sustaining relaxation behaviors, the idea of a growth mindset has been extensively studied and is associated with reduced anxiety and higher performance levels among students [13,32,33]. Growth mindset concepts could help students to overcome self-doubt about sustaining relaxation behavior. Furthermore, educators should encourage students to keep track of their participation in relaxation behaviors as a way to focus their emotions on behavior change. Students can then look back on this record of their progress as a way to overcome self-doubt in sustaining daily relaxation behaviors. Finally, interventions in the form of posters on campus or emails detailing the benefits of daily relaxation behaviors may help motivate students through the construct of emotional transformation.

In evaluating the results of this study, there are a few limitations to consider. First, the study’s cross-sectional design provides little more than a snapshot in time. Therefore, the results cannot be viewed temporally, and we do not technically know if the constructs predict behavior change. Secondly, as with any survey-based study, the self-reporting nature of the data lends itself to bias. While largely unavoidable, self-report bias must be considered in the analysis. Additionally, information obtained only reported students’ intentions to change behavior, not the end result of behavior change. A follow-up study could evaluate how well students’ actions align with their reported intentions. Furthermore, demographic data was mildly misrepresentative of the veterinary student demographic, preventing researchers from generalizing their findings to the entire veterinary student population. For example, while 92.9% of the students surveyed were female, the American Veterinary Medical Association (AVMA) reported in 2018 that females comprised only about 80% of the veterinary student population nationwide [34]. Finally, test–retest reliability was not performed for the study instrument and should be performed in all future MTM studies.

## 5. Implications for Practice and Future Research

This research study paves way for future interventional studies that will test the efficacy and effectiveness of MTM-based interventions for stress management among veterinary students. For conducting efficacy studies, since internal validity is of paramount importance, utilizing randomized controlled designs (RCTs) in small sample sizes will be appropriate. In designing such efficacy studies, the initiation construct of behavioral confidence will be specifically relevant. Mastery through small steps, role modelling, providing multifarious sources of confidence, and enhancing futuristic ability to perform stress-management behaviors may be fruitful methods of increasing behavioral confidence. For sustenance model, the emotional transformation construct will be especially relevant, in which interventions should incorporate educational methods such as psychodrama, role-play, and simulations that influence the affective aspects and help in directing feeling toward stress management behaviors. Once efficacy studies are able to support evidence derived from this cross-sectional study, effectiveness studies at different veterinary institutions with this target population can be designed.

## 6. Conclusions

Chronic stress among veterinary students and professionals is a growing issue in the field of veterinary medicine. Counselors and faculty members at veterinary schools have the ability to help students control stress levels by encouraging and providing resources for the use of conscious relaxation behaviors. The multi-theory model of health behavior change provides a starting point for interventions in veterinary schools. Based on the results of this study, interventions regarding conscious relaxation behaviors should target the constructs of behavioral confidence and emotional transformation. By focusing on veterinary student behavior, interventions will not only improve students’ experiences in school but will equip students with tools to use after graduation in combatting pitfalls in the veterinary profession such as compassion fatigue and burnout.

## Figures and Tables

**Table 1:** Socio-demographic Characteristics of the Participants (*N* = 140).

Age (Years)	Mean (SD)	*n* (%)
	25.88 (3.75)	
**Gender**		
Male		10 (7.1%)
Female		130 (92.9%)
**Race/Ethnicity**		
White or Caucasian American		123 (87.9%)
Black or African American		1 (0.7%)
American Indian or Alaska Native		1 (0.7%)
Asian American		4 (2.9%)
Hispanic American		6 (4.3%)
Other		5 (3.6%)
**Academic Classification**		
First year veterinary student		35 (25.0%)
Second year veterinary student		43 (30.7%)
Third year veterinary student		37 (26.4%)
Fourth year veterinary student		25 (17.9%)
**Grade Point Average**		
2.00–2.49		11 (7.9%)
2.50–2.99		27 (19.3%)
3.0–3.49		67 (47.9%)
3.50–4.00		34 (24.3%)
**Marital Status**		
Married		25 (17.9%)
Single		112 (80.0%)
Other		3 (2.1%)
**Children**		
Yes		6 (4.3%)
No		134 (95.7%)
**Work Status**		
Yes		16 (11.4%)
No		124 (88.6%)
**Living Arrangements**		
On-Campus		7 (5.0%)
Off-campus		133 (95.0%)

**Table 2:** Descriptive Statistics of Constructs of Multi-Theory Model (MTM) (*N* = 140).

Constructs	Possible Range	Observed Range	Mean (SD)	Cronbach’s Alpha
Intent for Initiation	0–4	0–4	0.95 (0.97)	–
Participatory Dialogue: Advantages	0–20	0–20	11.72 (3.95)	0.91
Participatory Dialogue: Disadvantages	0–20	0–20	10.13 (3.71)	0.75
Participatory Dialogue: Advantages−Disadvantages Score	−20–+20	−14–+14	1.59 (6.09)	–
Behavioral Confidence	0–20	0–12	3.33 (3.62)	0.88
Changes in Physical Environment	0–12	0–12	4.51 (3.54)	0.88
Entire Initiation Scale	–	–	–	0.79
Intent for Sustenance	0–4	0–4	0.79 (0.83)	–
Emotional Transformation	0–12	0–12	2.95 (2.86)	0.91
Practice for Change	0–12	0–9	1.77 (2.17)	0.74
Changes in Social Environment	0–12	0–12	4.67 (3.22)	0.77
Entire Sustenance Scale	–	–	–	0.87
Entire Scale	–	–	–	0.89

**Table 3:** Hierarchical Multiple Regression Predicting Initiation for Intentional Relaxation Behavior (*N* = 140).

Variables	*Unstandardized Coefficient*	*SE*	*Standardized Coefficient*	*p*-Value	95% *CI*
**Model 1**					
Perceived Stress	−0.009	0.015	−0.063	0.530	−0.038, 0.020
Depression ^a^	−0.413	0.199	−0.210	0.040	−0.807, −0.020
Academic Classification ^b^	−0.597	0.154	−0.319	<0.001	−0.901, −0.293
*F*(3, 125) = 7.902, *p* < 0.001, *R*^2^ = 0.159, Adjusted *R*^2^ = 0.139					
**Model 2**					
Perceived Stress	0.015	0.013	0.104	0.240	−0.010, 0.041
Depression ^a^	−0.290	0.158	−0.147	0.069	−0.603, 0.023
Academic Classification ^b^	−0.158	0.131	−0.084	0.230	−0.417, 0.101
Participatory Dialogue: Advantages−Disadvantages Score	0.010	0.013	0.067	0.421	−0.015, 0.035
Behavioral Confidence	0.138	0.021	0.540	<0.001	0.097, 0.179
Changes in Physical Environment	0.038	0.020	0.143	0.068	−0.003, 0.078
*F*(6, 122) = 19.929, *p* < 0.001, *R*^2^ = 0.495, Adjusted *R*^2^ = 0.470, Δ*R*^2^ = 0.336, Δ*F* = 27.022					

^a^ Reference category = no depression; ^b^ Reference category = first/second year veterinary students; SE = standard error of the unstandardized coefficient; 95% CI = 95% confidence interval for the unstandardized coefficient.

**Table 4:** Hierarchical Multiple Regression Predicting Sustenance for Intentional Relaxation Behavior (*N* = 140).

Variables	*Unstandardized Coefficient*	*SE*	*Standardized Coefficient*	*p*-Value	95% *CI*
**Model 1**					
Perceived Stress	−0.035	0.013	−0.272	0.009	−0.061, −0.009
Depression ^a^	−0.098	0.176	−0.057	0.579	−0.447, 0.251
Academic Classification ^b^	−0.454	0.135	−0.274	0.001	−0.722, −0.186
*F*(3, 125) = 8.323, *p* < 0.001, *R*^2^ = 0.166, Adjusted *R*^2^ = 0.146					
**Model 2**					
Perceived Stress	−0.005	0.011	−0.041	0.625	−0.027, 0.016
Depression ^a^	−0.045	0.142	−0.026	0.750	−0.325, 0.235
Academic Classification ^b^	−0.167	0.111	−0.101	0.134	−0.386, 0.052
Emotional Transformation	0.178	0.029	0.605	<0.001	0.120, 0.236
Practice for Change	0.026	0.036	0.068	0.478	−0.046, 0.097
Changes in Social Environment	−0.001	0.019	−0.004	0.959	−0.038, 0.036
*F*(6, 122) = 20.655, *p* < 0.001, *R*^2^ = 0.504, Adjusted *R*^2^ = 0.480, Δ*R*^2^ = 0.337, Δ*F* = 27.661					

^a^ Reference category = no depression; ^b^ Reference category = first/second year veterinary students; SE = standard error of the unstandardized coefficient; 95% CI = 95% confidence interval for the unstandardized coefficient.

## Data Availability

The datasets used and/or analyzed during the current study are available from the corresponding author on reasonable request.
